# Transcriptome of pancreas-specific *Bmpr1a*-deleted islets links to TPH1–5-HT axis

**DOI:** 10.1242/bio.011858

**Published:** 2015-07-17

**Authors:** Fang-Xu Jiang, Yuji Mishina, Akma Baten, Grant Morahan, Leonard C. Harrison

**Affiliations:** 1 The Walter & Eliza Hall Institute of Medical Research, 1G Royal Parade, Parkville, Victoria 3050, Australia; 2 Harry Perkins Institute of Medical Research, Centre for Medical Research, University of Western Australia, Nedlands, Western Australia 6009, Australia; 3 Department of Biologic and Materials Sciences, School of Dentistry, University of Michigan, Ann Arbor, MI 48109, USA

**Keywords:** BMPR1A, Transcriptome, Glucose homeostasis, Tph1

## Abstract

Bone morphogenetic protein (BMP) signaling is crucial for the development and function of numerous organs, but its role on the function of pancreatic islets is not completely clear. To explore this question, we applied the high throughput transcriptomic analyses on the islets isolated from mice with a pancreas-specific deletion of the gene, *Bmpr1a*, encoding the type 1a BMP receptor. Consistently, these pBmpr1aKO mice had impaired glucose homeostasis at 3 months, and were more severely affected at 12 months of age. These had lower fasting blood insulin concentrations, with reduced expression of several key regulators of β-cell function. Importantly, transcriptomic profiling of 3-month pBmpr1aKO islets and bioinformatic analyses revealed abnormal expression of 203 metabolic genes. Critically among these, the tryptophan hydroxylase 1 gene (*Tph1*), encoding the rate-limiting enzyme for the production of 5-hydroxytryptamine (5-HT) was the highest over-expressed one. 5-HT is an important regulator of insulin secretion from β cells. Treatment with excess 5-HT inhibited this secretion. Thus our transcriptomic analysis links two highly conserved molecular pathways the BMP signaling and the TPH1–5-HT axis on glucose homeostasis.

## INTRODUCTION

Bone morphogenetic proteins (BMP) are members of the transforming growth factor-β (TGFβ) super family (Derynck and Zhang, 2003). They are bound by heteromeric complexes of type 1 and type 2 serine/threonine kinase receptors, including BMP receptor type 1A (BMPR1A) and BMPR2 (Kishigami and Mishina, 2005). The BMP ligand/receptor complex phosphorylates intracellular signaling molecules SMAD1, 5 and 8 proteins. This in turn activates transcription factor SMAD-dependent and -independent pathways (Waite and Eng, 2003) that regulate targeted gene expression in multiple organs (Derynck and Zhang, 2003; Nohe et al., 2004) including the pancreatic islets for glucose metabolism (Goulley et al., 2007).

However, the molecular mechanism of BMP signaling on pancreatic islet function is poorly understood. Deletion of both *Bmpr1a* alleles in mice leads to developmental failure at the gastrulation stage (Mishina et al., 1995), precluding studies of this signaling in pancreas function. We previously demonstrated that global heterozygous *Bmpr1a*-deleted mice displayed abnormal glucose homeostasis, but the role of other insulin-sensitive tissues including the liver, skeletal muscle and adipose tissue could not excluded (Scott et al., 2009). To define specific functions of BMPR1A signaling in pancreatic islet cells, the Cre/loxP gene knockout strategy was employed. Reduction of BMPR1A signaling mediated by Cre recombinase under the control of the rat insulin promoter (RIP) was shown to induce diabetes from 2–3 months of age (Goulley et al., 2007). However, the direct linkage by which BMPR1A regulates glucose homeostasis was not described (Goulley et al., 2007).

We hypothesize that transcriptomic analysis of *Bmpr1a*-deleted islets would shed light on this mechanism. To test this hypothesis, we generated mice heterozygous or homozygous for pancreas-specific knockout of *Bmpr1a* (referred hereafter as pBmpr1aHet or pBmpr1aKO) in which the floxed *Bmpr1a* sequence was deleted by the expression of *Cre* transgene under the control of the promoter for *Pdx1* (the pancreas and duodenum transcription factor 1, also in humans called *IPF1* or *IDX1*) gene*.* Global gene expression and bioinformatics analyses reveal an unidentified molecular mechanism for BMP signaling on glucose homeostasis with another well conserved signaling pathway.

## RESULTS

### Impaired glucose homeostasis in pBmpr1aKO mice

**Fig. 1.**
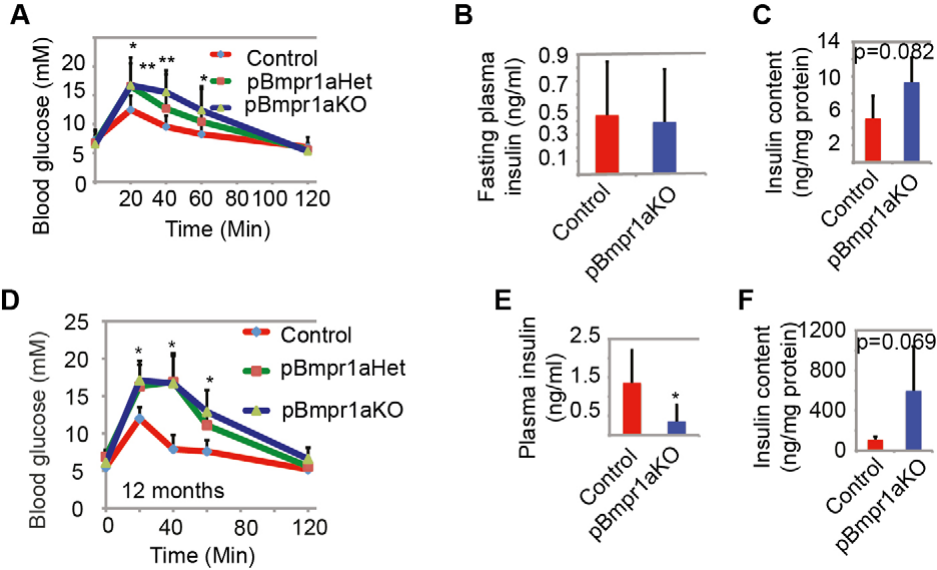
**Impaired glucose** **homeostasis** **in pBmpr1aKO mice.** (A) Blood glucose profiles at 3 months of age. After fasting for 12 h, tail vein blood was taken to measure blood glucose concentrations just before and at 20, 40, 60 and 120 min after intraperitoneal glucose (1 g/kg). Mean±s.d., *P<0.05, **P<0.01 compared to Control. (B) Plasma insulin profiles. Fasting insulin concentrations were determined by ELISA in 3-month male mice. Mean±s.d., *n*=6. (C) Pancreas insulin content profiles. Pancreas was dissected and its insulin content was acid-extracted and determined by ELISA in 3-month male mice. Mean±s.d., *n*=6. (D) Blood glucose profiles in 12-month male mice. After fasting for 12 h, tail vein blood was taken to measure blood glucose concentrations just before and at 20, 40, 60 and 120 min after intraperitoneal glucose (1 g/kg). Mean±s.d., *P<0.05, *n*=15/group. (E) Plasma insulin profiling in 12-month male mice. Fasting insulin concentrations were determined by ELISA. Mean±s.d., *P<0.05, *n*=8/group. (F) Pancreas insulin content profiling in 12-month male mice. Pancreas was dissected and its insulin content was determined by ELISA. Mean±s.d., *n*=5/group.

The strategy to generate pBmpr1aKO mice was summarized in supplementary material Fig. S1. The postnatal development of body and pancreas masses was similar between Control and pBmpr1aKO mice in various time-points between 7 and 20 weeks of age (supplementary material Fig. S2A,B). As *Pdx1-dnBmpr1a* and insulin promoter-derived *Bmpr1a*-deleted mice develop diabetes at 2–3 months of age (Goulley et al., 2007), we also performed intraperitoneal glucose challenge in our pBmpr1aKO mice at 3 months of age. Compared to Control mice, pBmpr1aKO and to a less extent pBmpr1aHet mice had significantly higher blood glucose concentrations from 20 to 60 min (Fig. 1A). Although their fasting plasma insulin levels were not significantly different (Fig. 1B), pancreatic insulin content was modestly increased in pBmpr1aKO islets (Fig. 1C).

Similarly, at 12 months of age, blood glucose concentrations at 20, 40 and 60 min after intraperitoneal glucose challenge were significantly higher in pBmpr1aKO mice than in Control mice (Fig. 1D). In 10/23 pBmpr1aKO mice, blood glucose was >20 mM at 20–40 min. Consistent with more severely impaired glucose homeostasis, the fasting plasma insulin concentration in 12-month old pBmpr1aKO mice was significantly lower and total pancreatic insulin content was up to 10-fold higher (but not significantly different due to a high individual variability) than that in Control mice (Fig. 1E,F). These data were largely consistent with, though milder than, those previously reported (Goulley et al., 2007).

### Decreased expression of PDX1 and GLUT2 in aging pBmpr1aKO islets

**Fig. 2.**
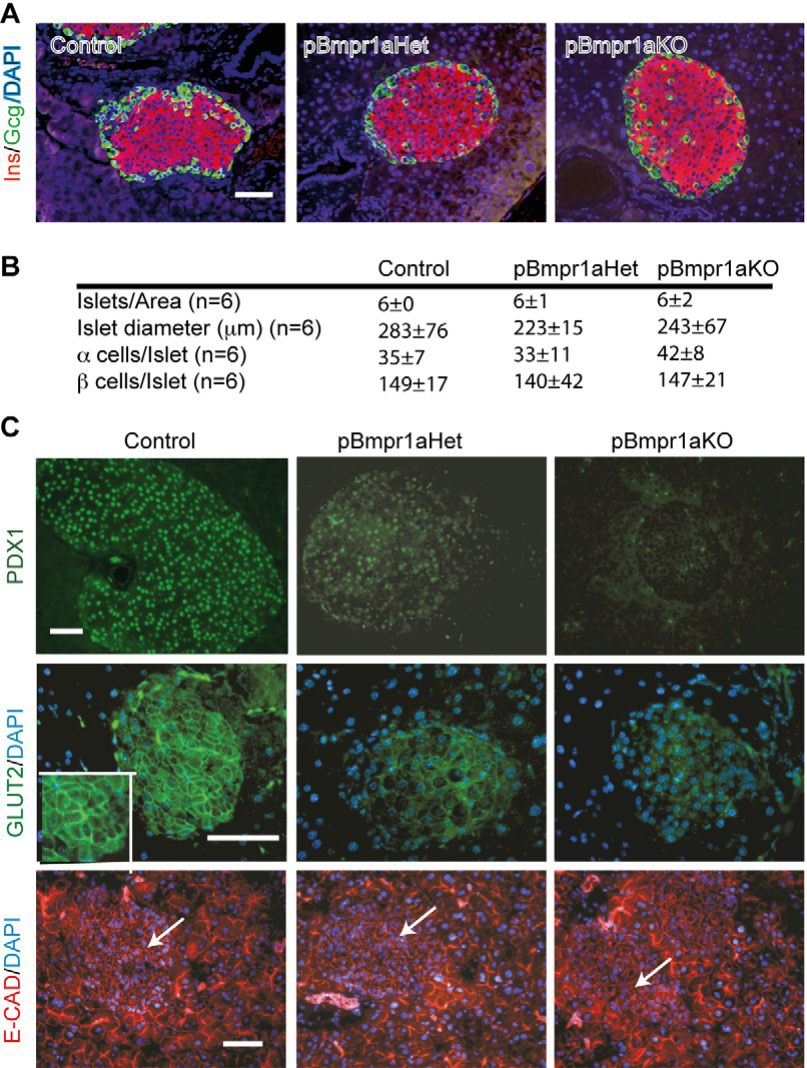
**Reduced expression of PDX1 and GLUT2 in pBmpr1aKO islets in 12 month-old mice.** (A) Immunofluorescence analyses. Representative images show co-staining with insulin (INS, red) and glucagon (Gcg, green) in Control, pBmpr1aHet and pBmpr1aKO pancreas. Scale bar=50 µm. (B) Morphometric analyses showing islet number and diameter and numbers of α and β cells in all three genotypes. (C) Immunofluorescence analyses. Representative images show staining for PDX1 (green), GLUT2 (green) and E-cadherin (E-CAD, red) after counterstaining with the DNA dye DAPI (blue) (arrow, islet). The inset represents a higher magnification of the islet area. Scale bars=50 µm.

As glucose homeostasis can be impaired by inadequate numbers and ratios of endocrine cells (Dickson and Rhodes, 2004; Unger and Orci, 2010), islet architecture and the numbers of α and β cells at 12 months of age were therefore determined. Immunofluorescence staining and morphometric analyses showed that the distribution of α and β cells was similar and their number were not significantly different among all three genotypes (Fig. 2A,B).

As PDX1 activates key genes including *Ins* and *Glut2* (Ohneda et al., 2000) for functional β cells (Holland et al., 2005), its expression was examined. Antibodies against PDX1 and glucose transporter-2 (GLUT2) stained strongly the nuclei and cell membrane of Control islets, but weakly and very faintly of pBmpr1aHet and pBmpr1aKO islets, respectively (Fig. 2C). In contrast, in all three genotypes the expression of E-CAD was strong in exocrine and ductal cells but visible and unchanged in islet cells (Fig. 2C).

### Transcriptomic analyses of BMP signaling genes in pBmpr1aKO islets

**Fig. 3.**
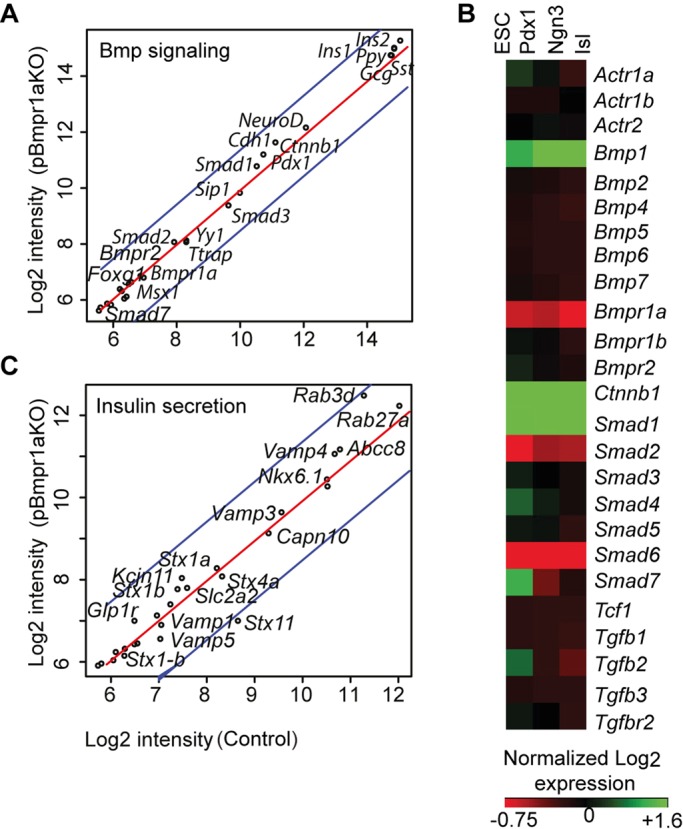
**Comparable low expression of BMP signaling genes in Control and pBmpr1aKO islets.** Performed and profiled on RNA extracted from purified islets at 3 months in Control and pBmpr1aKO mice, and annotated in the genome-wide expression datasets. (A) Pairwise scatter gene expression profiles showing a large array of genes for selective BMP signaling and hormones. Blue lines indicate a two-fold difference in expression values. Gene expression levels are exhibited as log_2_ intensities. (B) Gene expression heatmap. The map is generated as described previously (Sui et al., 2012) by annotation of publically available global expression raw datasets (Gu et al., 2004; Hirst et al., 2006). The heatmap shows a dynamic gene expression pattern of selected BMP and TGFβ signaling genes containing three contrasts of four developmental stages including embryonic stem cells (ESC), purified pancreatic progenitors (Pdx1) and islet progenitors (Ngn3), and isolated adult islets (Isl). (C) Pairwise scatter gene expression profiles showing a large array of genes for selective insulin processing and secretion pathways.

To identify potential molecular linkages of how perturbation of BMPR1A signaling in the pancreas impairs glucose homeostasis, we purified Control and pBmpr1aKO islets at 3 months of age for RNA extraction and global transcriptomic analysis. The microarray chips we used contained 46,657 probes each, covering almost all known protein-encoding genes. As expected, gene annotation and bioinformatics pairwise scatterplot analyses showed that the two genotypes had comparable expression of numerous genes. *Bmpr1a* expression was equivalent at a low level in both Control and pBmpr1aKo islets, as the two probes in our transcriptomic chips were not targeted the sequence encoded by the deleted “Exon 4” (Fig. 3A). Surprisingly, however, the expression of many other BMP signaling genes (*Bmpr2*, *Smad1*, *Smad2*, *Smad3* and *Smad7*) and down-stream SMAD interacting molecule genes (*Sip1*, *Yy1*, *Trap* and *Msx1*) also was low and unchanged, with reference to medium to high expression of genes encoding key β-cell markers (PDX1 and NEUROD) and hormones (INS1, INS2, GCG, SST and PPY) (Fig. 3A). To shed potential light on why the expression of many BMP and other TGFβ signaling genes was low and unchanged at this stage, we analyzed publicly available purified population-derived microarray datasets (Gu et al., 2004; Hirst et al., 2006) for the developmental pattern of most these genes. We found many of such genes (*Actr1a*, *Bmp1*, *Bmp2*, *Bmp4*, *Bmpr1a*, *Smad4*, *Smad7*, *Tgfb2* and *Tgfbr2*) were differentially expressed during pancreas specification, becoming progressively down-regulated during islet cell development and function (Fig. 3B).

### Absence of abnormal expression of classical genes regulating insulin secretion

In further searching for the molecular linkage of how deletion of *Bmpr1a* in the pancreas impaired glucose homeostasis, we mined our transcriptomic dataset for genes encoding molecules for classical insulin processing and secretion (RAB27A, RAB3D, ABCC8, VAMP4, VAMP3, CAPN10, STX1A, STX4A, KCJN11, SLC2A2, STX1B, GLP1R, STX1-B and STXBP3). Interestingly, we noted that the expression of these genes though in various ranges was unchanged in pBmpr1aKO compared to Control islets (Fig. 3C). Taken together, the data suggest that impaired glucose metabolism in pBmpr1aKO mice may be due to abnormal expression of genes that encode molecules in other unidentified molecular pathway(s), rather than the well-known regulators of insulin processing and secretion.

### Abnormal expression of 203 metabolic genes in pBmpr1aKO islets

**Fig. 4.**
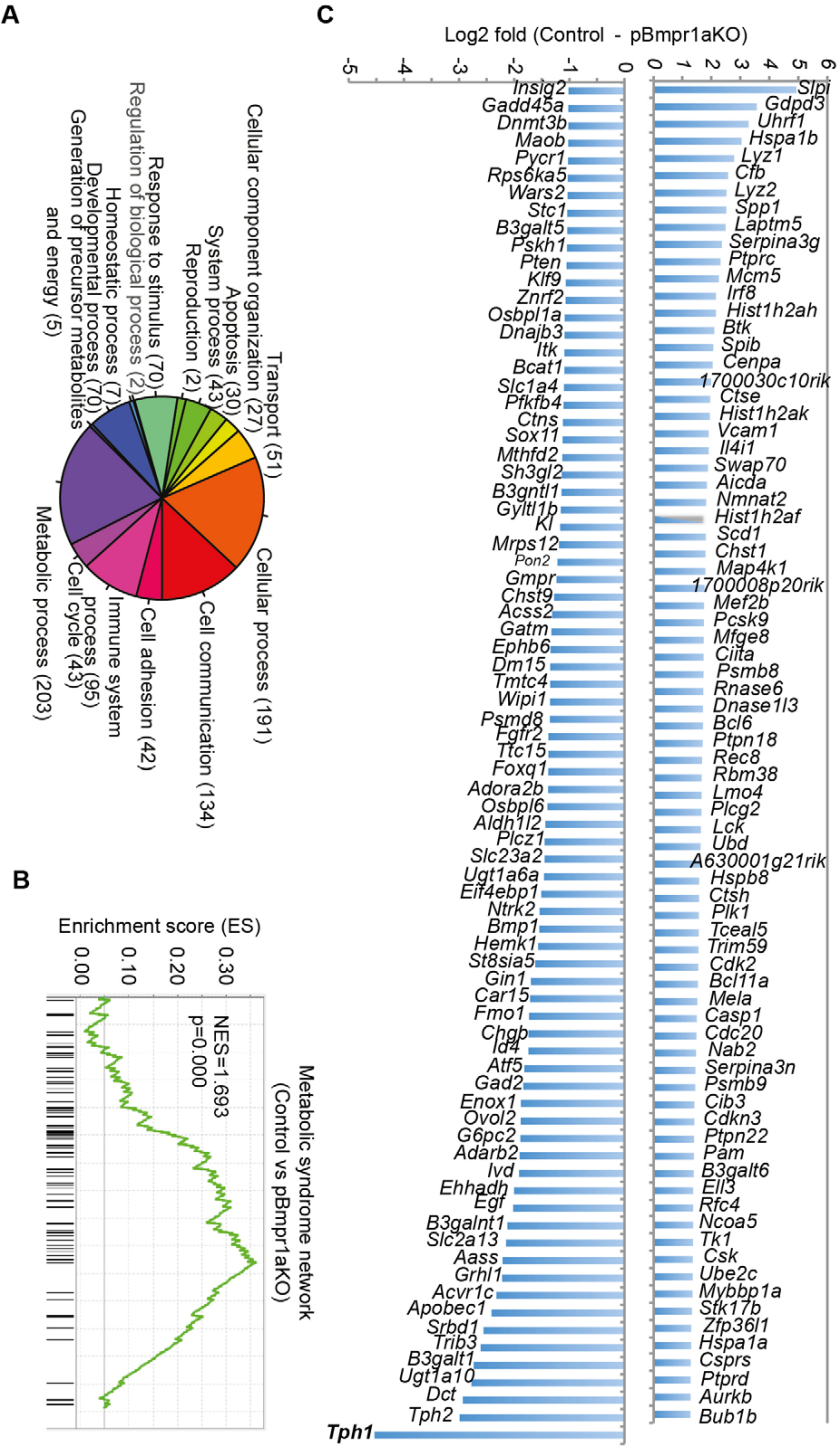
**Abnormal expression of metabolic genes in pBmpr1aKO islets.** (A) Pie graph categorizing differentially expressed genes. The number of genes in each category is shown in parentheses. (B) Geneset enrichment analysis showing enrichment of the metabolic syndrome network. Normalized enrichment score (NES) was reported. (C) Core differentially expressed metabolic genes showing all down-regulated and log_2_>1.27 up-regulated genes.

Transcriptomic mining and bioinformatics analyses indeed identified that ∼700 genes involved in a variety of biological processes were up- or down-regulated over 2-fold (Fig. 4A), including genes encoding molecules associated with stress (ATF5 and RAD23A), transporters (CFTR, SLC27A2 and SLC6A8) and DNA replication (CCNB1, CDK, CDK2, CYCLIN B and D) (supplementary material Figs S3 and S4). Importantly among the 203 genes encoding molecules involved in metabolism, 125 were down-regulated (>2-fold) while 78 were up-regulated. Gene set enrichment analyses (Subramanian et al., 2005) revealed that a set of genes for metabolic syndrome network was enriched (Fig. 4B). Crucially, core differentially genes consisted of the most over-expressed *Tph1* (∼20-fold higher in pBmpr1aKO islet cells) and *Tph2* and the most down-regulated *Slpi* (encoding antileukoproteinase, an anti-inflammation molecule) (Eipel et al., 2007) (Fig. 4C).

### High over-expression of *Tph1* in pBmpr1aKO islets

**Fig. 5.**
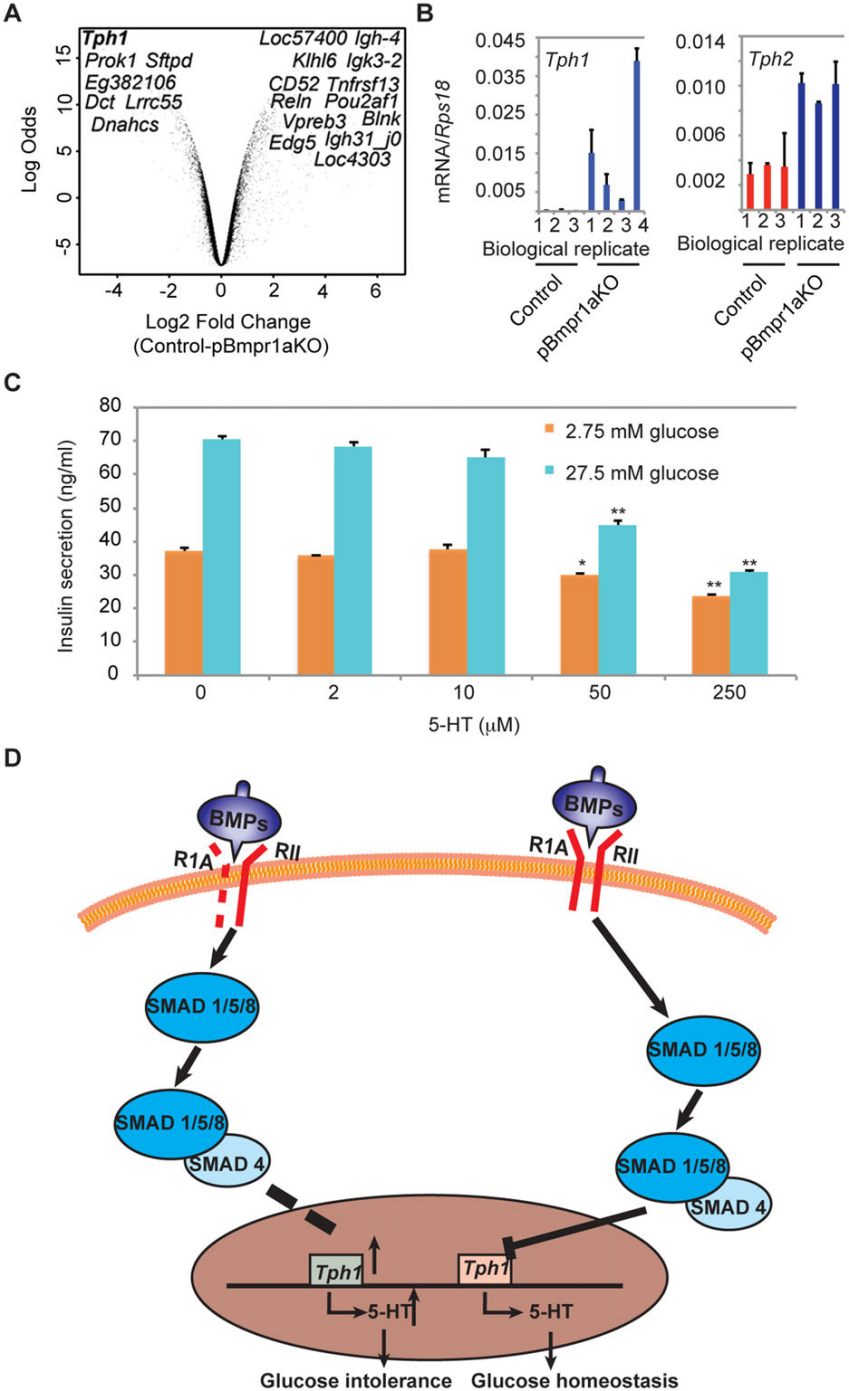
**Chronic over-expression of** ***Tph1* in pBmpr1aKO islets and impaired insulin secretion.** (A) Volcano plots showed most significantly up- and down-regulated genes amongst the 203 metabolic genes. (B) Real time RT-PCR analysis of *Tph1* and *Tph2* performed on cDNA reverse transcribed from RNA extracted from purified islets at 3 months in Control and pBmpr1aKO mice. Mean±s.d. (C) Analyses of glucose-stimulated insulin secretion performed on MIN6 cells after treatment with various doses of 5-HT as indicated. Mean±s.d., *P<0.05 and **P<0.01 vs 0 µM 5-HT respectively, *n*=3. (D) Diagram showing how BMP signaling links to the TPH1–5-HT pathway for glucose homeostasis. Whereas normally BMP signaling negatively regulates the TPH1–5-HT pathway, dashed lines indicate that when it was deleted, *Bmpr1a* suppression on *Tph1* was diminished or disappeared, thus *Tph1* and *5-HT* overexpressed, and glucose homeostasis impaired.

Unlike *Slpi*, Volcano plot analysis confirmed that *Tph1* was the most over-expressed gene (Fig. 5A), suggesting that *Tph1* and *Tph2* might be novel mediator genes of BMPR1A signaling. To verify the over-expression of *Tph1* and *Tph2*, islets were isolated from Control and pBmpr1aKO mice at 3 months of age at which transcriptomic analysis was performed. Analyses by qRT-PCR demonstrated that though individually variable, *Tph1* and *Tph2* were up-regulated by at least 100- and 2-fold in pBmpr1aKO islets, respectively (Fig. 5B). It is well documented that over-expression of *Tph1* and *Tph2* parallels the increase of TPH1 and 5-HT content in β-cell granules (Kim et al., 2010; Schraenen et al., 2010), so the gene over-expression in pBmpr1aKO islets would contribute to the abnormal accumulation of TPH1 and 5-HT. This in turn would suggest that the abnormal excess of 5-HT in pBmpr1aKO islets might link to the impaired glucose homeostasis in the pBmpr1aKO mice.

### Excess 5-HT impaired insulin secretion in β cells

To test the hypothesis that excess 5-HT indeed impairs insulin secretion in β cells, ideally we could use purified Control β cells, however, lack of their specific marker prevented us from doing so. Instead, we tested the effect of excess 5-HT on the function of the clonal β-cell line, MIN6 (Miyazaki et al., 1990). MIN6 cells were treated with several concentrations of 5-HT for 4 days and then quantitated the glucose-stimulated insulin secretion (GSIS). In response to 5-HT treatment, these cells, whereas were morphologically unchanged, displayed dose-dependent impairment on GSIS, and the latter was reduced up to 60% after treatment with 250 µM 5-HT (Fig. 5C). These data supported the notion that excess 5-HT inhibits insulin secretion in β cells (Fig. 5D) and corroborated with our transcriptomic analysis.

## DISCUSSION

Our transcriptomic analysis of *Bmpr1a*-deleted islets identifies a previously unidentified linkage of two highly conserved signaling pathways on glucose homeostasis, namely the BMP-BMPR1A and the TPH1–5-HT cascades. Interestingly, foregut-specific deletion of *Bmpr1a* increased an approximately 3-fold of 5-HT-expressing endocrine cells in the intestine (Maloum et al., 2011). Hence BMPR1A negatively regulates the 5-HT signaling system not only in islets but also in other neuroendocrine systems.

Although its up-stream regulator(s) remained unknown, the role of TPH1–5-HT system on glucose homeostasis has been well documented. Treatment of 500 µM–100 mM 5-HT to isolated mouse (Gagliardino et al., 1974; Paulmann et al., 2009) or rat islets (Zawalich et al., 2001, 2004) or golden hamster pancreas (Feldman and Lebovitz, 1970) reduced GSIS by ∼15-100%. Similarly, administration of pharmacological doses of 5-HT *in vivo* leads to hyperglycemia (Paulmann et al., 2009). Treatment with a 5-HT receptor 2c (5-HT_2C_R) antagonist significantly increases insulin secretion in islets of the db/db mice (Zhang et al., 2013), a model of type 2 diabetes (T2D). Clinically, plasma 5-HT level in people with T2D, a pandemic metabolic disorder, is increased (Hara et al., 2011), although it is unknown whether it plays a contributing role in, or is the consequence of, this disease. Nevertheless, these data did not demonstrate that the TPH1–5-HT signaling in β cells regulates glucose homeostasis although previous studies had identified 5-HT packaged into the insulin granules (Ekholm et al., 1971; Jaim-Etcheverry and Zieher, 1968) and a convergence of the insulin and 5-HT programs in islet β cells (Kim et al., 2010; Ohta et al., 2011). In combining MIN6 cell data, our findings suggest that BMPR1A signaling regulates glucose metabolism via suppressing β-cell TPH1–5-HT axis. Future studies are required to explore other potential mechanisms including stress on glucose homeostasis.

Nevertheless, deficiency of TPH1–5-HT signaling in β cells also impairs glucose homeostasis. Mice with homozygous deletion of the serotonergic transcription factor gene *Fev*, critical for the production of the TPH1–5-HT system, had affected insulin gene expression, and impaired insulin secretion and glucose tolerance (Ohta et al., 2011). In *Tph1* knockout mice, the concentration of islet 5-HT was reduced by 10-fold, and diabetes developed as early as 14 days after birth (Paulmann et al., 2009). Furthermore, genetic deletion of *5-HT_2b_R* or *5-HT_2c_R* also impaired glucose tolerance (Kim et al., 2010; Nonogaki et al., 1998).

In addition to the BMP signaling, the TPH1–5-HT axis is regulated by lactogenic signaling in pregnant islet β cells. Previous reports demonstrate that TPH1 and 5-HT are transiently up-regulated in these cells by lactogens (Kim et al., 2010; Schraenen et al., 2010). This up-regulation stimulates the proliferation of pregnant β cells (Kim et al., 2010) and improves their GSIS (Ohara-Imaizumi et al., 2013) to accommodate a high-energy demand. Furthermore, pregnant but not non-pregnant mice with homozygous deletion of *Htr3a* displayed impaired glucose homeostasis and their isolated islets though proliferating normally lacked the pregnant GSIS (Ohara-Imaizumi et al., 2013). Taken together, these data demonstrate that under the control of different up-stream regulators, the complex TPH1–5-HT-receptor system is operated in β cells for glucose metabolism in physiological and pathophysiological conditions.

Finally, our transcriptomic analysis suggests that BMPR1A plays a negative role in adult islets. We showed that the expression of *Bmpr1a* and selective TGFβ superfamily genes was generally low at 3 months of age; implying that BMPR1A signaling is more important in developing than in adult islets. More severe glucose intolerance at 12 months might be due to an age effect (Reaven, 2003) on the basis of 3 months of age. Our data also provide an at least partial molecular explanation why treatment with several selected BMP and other TGFβ superfamily ligands do not show any significant effect on isolated adult C57BL/6 islets (Brown et al., 2011). In contrast, over-expression of *Bmp4* in β cells or systemic administration of BMP4 has been reported to enhance GSIS (Goulley et al., 2007). Moreover, the *Pdx1-dnBmpr1a* mice had significantly lower expression of many genes, involved in insulin production, processing and secretion, in addition to those for BMPR1A signaling (Goulley et al., 2007). The reasons for the discrepancy among these studies were not completely clear but may be explained as follows. First, the genetic background difference may lead to the phenotypic difference: whereas our *Bmpr1a^flox/flox^* and *Pdx1-Cre* mice have been bred on the C57BL/6 background for over 10 generations, the *Pdx1-dnBmpr1a* mice are generated on a mixture of C57BL/6 and CBA genetic backgrounds (Goulley et al., 2007). Second, the RIP-Cre mouse line used by Goulley et al. (2007) was reported to exhibit glucose intolerance without crossing with any floxed line (Lee et al., 2006).

In summary, our transcriptomic analysis of *Bmpr1a*-deleted islets links two crucial regulatory pathways for glucose homeostasis. Establishing in detail the mechanism through which BMPR1A suppresses the expression of *Tph1* and via 5-HT to regulate glucose homeostasis would generate new knowledge for pathogenesis, diagnosis and drug targets of the pandemic T2D.

## MATERIALS AND METHODS

### Generation of conditional Bmpr1a-deleted mice

The *Pdx1-Cre* transgene used has been previously well-characterized (Heiser et al., 2006; Herrera, 2000) and becomes active around E11.5 (Heiser et al., 2006). Heterozygous Cre mice under the control of *Pdx1* promoter (*Pdx1-Cre*) (Herrera, 2000) and homozygous mice carrying floxed alleles for the fourth coding exon of *Bmpr1a* (*Bmpr1a^flox/flox^*) (Mishina et al., 2002) were bred on the C57BL/6 background for at least 10 generations. In order to generate a compound heterozygous biogenic animal heterozygous for transgenic *Pdx1-Cre* and for the floxed *Bmpr1a* allele (*Bmpr1a^flox/+^*), namely *Pdx1-Cre; Bmpr1a^flox/+^* (known as pBmpr1aHet hereafter), the *Pdx1-Cre* mice were bred to the *Bmpr1a^flox/flox^* mice. The pBmpr1aHet mice were then crossed with the *Bmpr1a^flox/flox^* mice to generate homozygous conditional *Bmpr1a*-deleted (*Pdx1-Cre*; *Bmpr1a^flox/flox^*, namely pBmpr1aKO) mice, as well as mice with three other distinct genotypes: pBmpr1aHet, *Bmpr1a^flox/+^* and *Bmpr1a^flox/flox^*. The latter two genotypes as well as *Pdx1-Cre* mice were phenotypically normal (Ahn et al., 2001) and used as the control (designated as Control hereafter). All mice were maintained for a 12:12 h light and dark cycle and fed with normal chew. PCR of tail and pancreas DNA was used to genotype the progeny (supplementary material Fig. S1A). PCR primers are described elsewhere (Mishina et al., 2002). Deletion of *Bmpr1a* exon 4 has been previously shown to be sufficient for inactivation of BMPR1A function (Mishina et al., 2002). To confirm *Cre*-mediated DNA recombination of *Bmpr1a*, southern blot analysis was performed on pancreatic tissue DNA. In Control pancreas, a 4.3 kb fragment of genomic DNA was detected, whereas in pBmpr1aKO after deletion of the *Bmpr1a* exon 4, only a 2.3 kb fragment was visible (supplementary material Fig. S1B), verifying exon 4 deletion and the efficacy of recombination. All experiments were performed in accordance with guidelines covering the care and use of animals in research, as approved by the Walter and Eliza Hall Institute of Medical Research Animal Ethics Committee.

### Histology and immunofluorescence studies

The pancreas of 3 and 12 month-old mice with Control, pBmpr1aHet and pBmpr1aKO genotypes were fixed in 4% paraformaldehyde in phosphate saline buffer (PBS) and processed for routine haematoxylin and eosin staining and for immunofluorescence histology.

Guinea pig antiserum to pig insulin and rabbit antisera to pig glucagon were from Dako (Glostrup, Denmark). Rat IgG2a mAb to E-cadherin (E-CAD) was from Invitrogen (Melbourne, Australia). Rabbit immunoglobulins (Ig) to mouse PDX1 was generated in-house (Holland et al., 2005) and from elsewhere (Suzue et al., 1989). Mouse glucose transporter 2 (GLUT2) was purchased from R&D Systems (Minneapolis, USA). FITC-conjugated streptavidin, Texas Red-conjugated goat anti-guinea pig Ig, FITC-conjugated rabbit anti-rat or sheep anti-rabbit Ig and Alexa 568-conjugated goat anti-rabbit Ig were purchased from Caltag Laboratories (Burlingame, USA), Vector Laboratories, (Burlingame, USA), Molecular Probes (Eugene, USA) and Chemicon International (Temecula, USA), respectively. The immune fluorescence staining was essentially as described previously (Jiang and Harrison, 2005). Microphotographs were taken under an inverted Olympus IX71 U-RFL-T fluorescent microscope with the same exposure time between samples.

### Morphometric analyses

The pancreases of 12 month-old mice with Control, pBmpr1aHet and pBmpr1aKO genotypes were fixed and processed for serial sections. Five sections per pancreas were randomly sampled as we described previously (Jiang et al., 1994) for glucagon and insulin staining and for measurements of islet diameters. The number of α and β cells was counted manually in images with original magnifications of 20× or 40× and verified with Image J software.

### Isolation of islets

Islets of Langerhans were isolated from Control and pBmpr1aKO male mice at 3 months of age as described previously (McKenzie et al., 2010). Briefly, the pancreas was injected via the bile duct with collagenase P solution (1.2 mg/ml dissolved in Hanks’ balanced salt solution containing 2 mM Ca^2+^ and 20 mM HEPES). Islets were isolated by density gradient centrifugation, washed and handpicked for RNA extraction.

### Real time quantitative RT-PCR (qRT-PCR) analyses

Primer sequences for *Tph1* and *Tph2* are 5′-cggatcagaagactcccagc-3′; 5′-tccgggactcgatgtgtaac-3′ and 5′-tctacaccccggaaccagat-3′; 5′-gcaaaggccgaactcgattg-3′, respectively. qRT-PCR analysis was essentially as we described recently (Jiang et al., 2010). Briefly, Power SYBR Green PCR Master Mix was from Applied Biosystems (Foster City, USA) or from Bioline (Sydney, Australia). cDNA was amplified by PCR: 95°C for 10 min, followed by 40 cycles of 95°C for 15 s and 60°C for 1 min. The number of cycles of threshold (Ct) was measured with an ABI Prism 7900HT Sequence Detection System (Applied Biosystems) or a Rotor-Gene RG-3000 (Corbet Research, Sydney, Australia). All quantifications were normalized with the internal 18s rRNA level (2^−ΔCt^). The specificity of each product was determined by its distinct dissociation curve.

### Genome-wide transcriptomic profiling

Total RNA was extracted from islets at 3 months old with Trizol reagent (Invitrogen) according to the manufacturer's instructions. The quality and concentration of the total RNA were determined by the Agilent Bioanalyzer 2100 system (Eukaryote Total RNA Nano, Agilent Technologies, Melbourne, Australia). Samples with RNA integrity number ≥8 were used for further experiments. Three independent samples were performed for each genotype. Each RNA sample was processed with the Illumina^®^ TotalPrep RNA Amplification Kit to produce labeled cRNA. The cRNA from each sample was then hybridized to an Illumina MouseRef-8 v1.1 Expression BeadChip (Illumina, San Diego, USA). Raw image data were generated with the Illumina Bead Scanner.

### Accession code

The raw transcriptomic datasets were stored at Gene Expression Omnibus (http://www.ncbi.nlm.nih.gov/geo) with an accession number of GSE41699.

### Bioinformatics analysis

Global profiling datasets were analyzed as described previously (Jiang et al., 2010; Sui et al., 2012). Briefly, after quality check, log_2_ transformation of raw datasets and inter-chip normalization, the differential expression of genes (*P*≤0.05; −1≤log_2_≥1) between Control and pBmpr1aKO islets was analyzed using the Limma package in the “R” environment (http://bioinf.wehi.edu.au/limma). Geneset enrichment analysis was performed (http://www.broadinstitute.org/gsea/msigdb/genesets.jsp). The differentially expressed datasets was also subjected to Ingenuity pathway analysis (www.ingenuity.com).

### Cell culture and glucose-stimulated insulin secretion

β-cell line MIN6 cells (Miyazaki et al., 1990) at passage 32 were cultured in low glucose DMEM supplemented with 2% B27 (Invitrogen), 100 μg/ml streptomycin, 100 units/ml penicillin and 2 mM glutamine in the presence of various concentrations of 5-HT (Sigma, Sydney, Australia) for 4 days with 10% CO_2_ at 37°C. After pre-incubation with Krebs-Ringer buffer (Seaberg et al., 2004) at 37°C for 90 min, the cells were incubated at 37°C for 60 min with basal (2.75 mM) or stimulus (27.5 mM) D-glucose, and conditioned media collected to determine insulin concentration (see below).

### Intraperitoneal glucose tolerance test and insulin assays

For the intraperitoneal glucose tolerance test, mice were fasted overnight (∼12 h) and tail vein blood was then collected just before and at 20, 40, 60 and 120 min after injecting glucose 1 gm/kg body weight intraperitoneally. Blood glucose concentration was measured by the glucose oxidase method with a portable glucometer (Roche Diagnostics, Mannheim, Germany). Plasma was separated from retro-orbital vein blood samples. Insulin was extracted with acid ethanol from minced pancreas. Plasma insulin concentrations and total pancreas insulin contents were measured with an Ultrasensitive ELISA Mouse Insulin kit (Mercodia AB, Uppsala, Sweden).

### Statistics

Differences between groups were analyzed by non-parametric, unpaired Mann–Whitney *U*-Tests or analysis of variance. Data are presented as mean±s.d. of 3–25 independent experiments.

## Supplementary Material

Supplementary Material
