# Supplementary material for: Transcriptome of pancreas-specific Bmpr1a-deleted islets links to TPH1–5-HT axis
Source: Biol Open. 2015 Jul 17;4(8):1016–23. doi: 10.1242/bio.011858 (PMC4542282; doi:10.1242/bio.011858)
Supplement: Supplementary Material [file supp_4_8_1016__index.html]

Transcriptome of pancreas-specific Bmpr1a-deleted islets links to TPH1–5-HT axis — Transcriptome of pancreas-specific Bmpr1a-deleted islets links to TPH1–5-HT axis — Supplementary Material 

# Transcriptome of pancreas-specific *Bmpr1a*-deleted islets links to TPH1–5-HT axis

## BIO011858 Supplementary Material

- Supplementary Material
