# Supplementary material for: Transcriptome of pancreas-specific Bmpr1a-deleted islets links to TPH1–5-HT axis
Source: Biol Open. 2015 Jul 17;4(8):1016–23. doi: 10.1242/bio.011858 (PMC4542282; doi:10.1242/bio.011858)
Supplement: Supplementary Material [file supp_bio.011858_BIO011858supp.pdf]

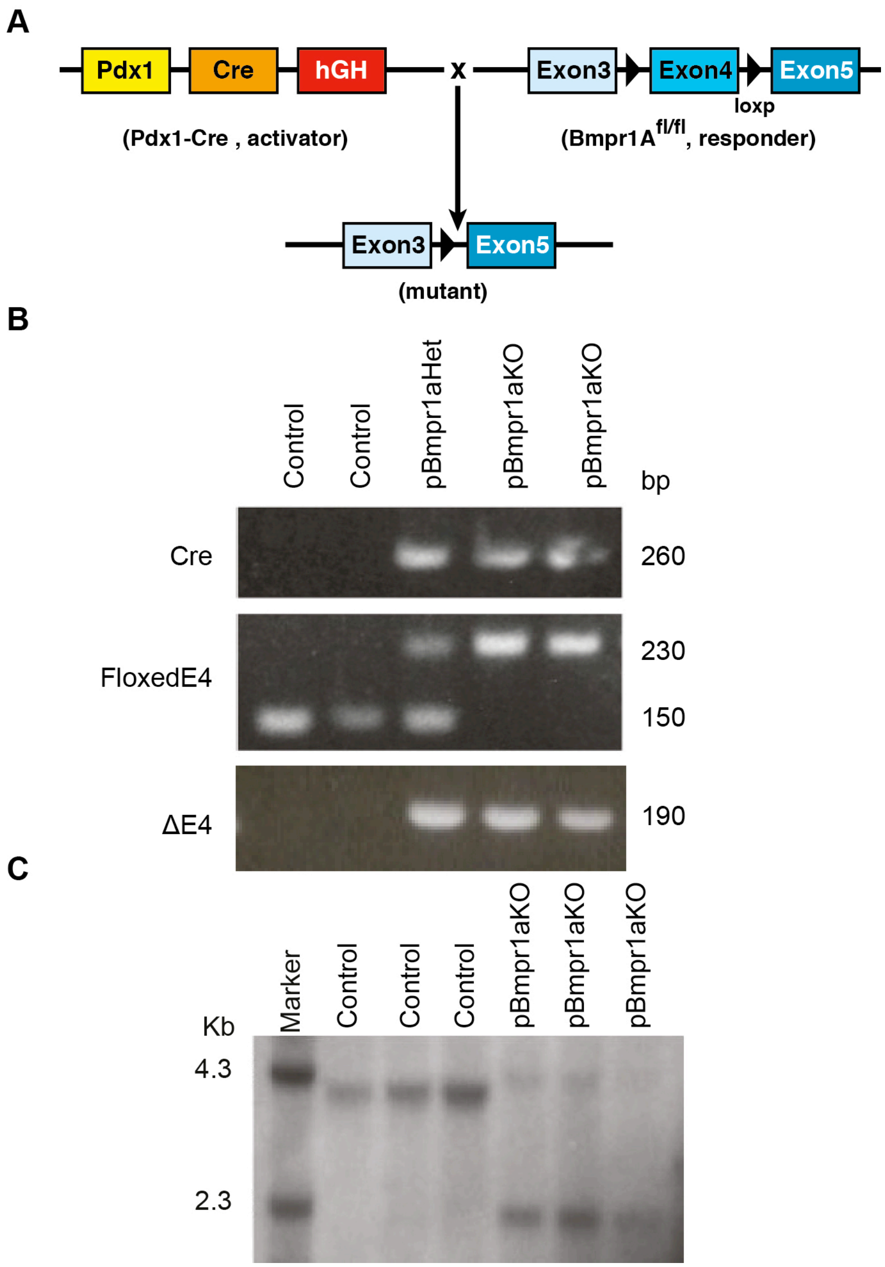

Fig. S1. Strategies for generating pancreas-specific deletion of *Bmpr1a* and for genotyping. (A) Schema of *Bmpr1a* exon-4 deletion. (B) PCR-based strategy for genotyping mutant mice. The 5' gcagctgctgctgcagcctcc 3'; 5' tggctacaatttgtctcatgc 3' primers were used to detect either a 150 Wildtype sequence or a 230 floxed sequence. The 5' ggtttggatcttaaccttagg 3'; 5' tggctacaatttgtctcatgc 3' primers were used to detect a product after the deletion of the floxed sequence, as described (Mishina et al., 1995). (C) Southern analysis of Cre-mediated deletion of *Bmpr1a*. Genomic DNA was purified from E19.5 pancreas and probed with H23 as described previously (Ahn et al., 2001).

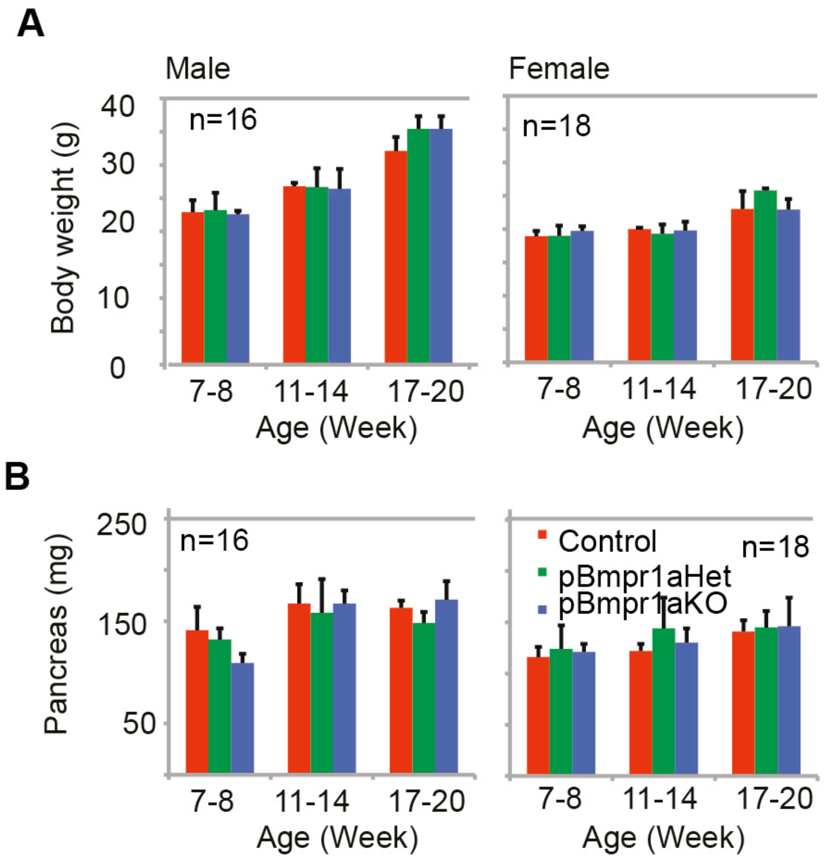

Fig. S2 Postnatal analyses of Control and pBmpr1aKO mice. Body and pancreas masses in male and female mice in a dynamic age range from 7 to 20 weeks with indicated numbers of mice studied. (mean±s.d.).

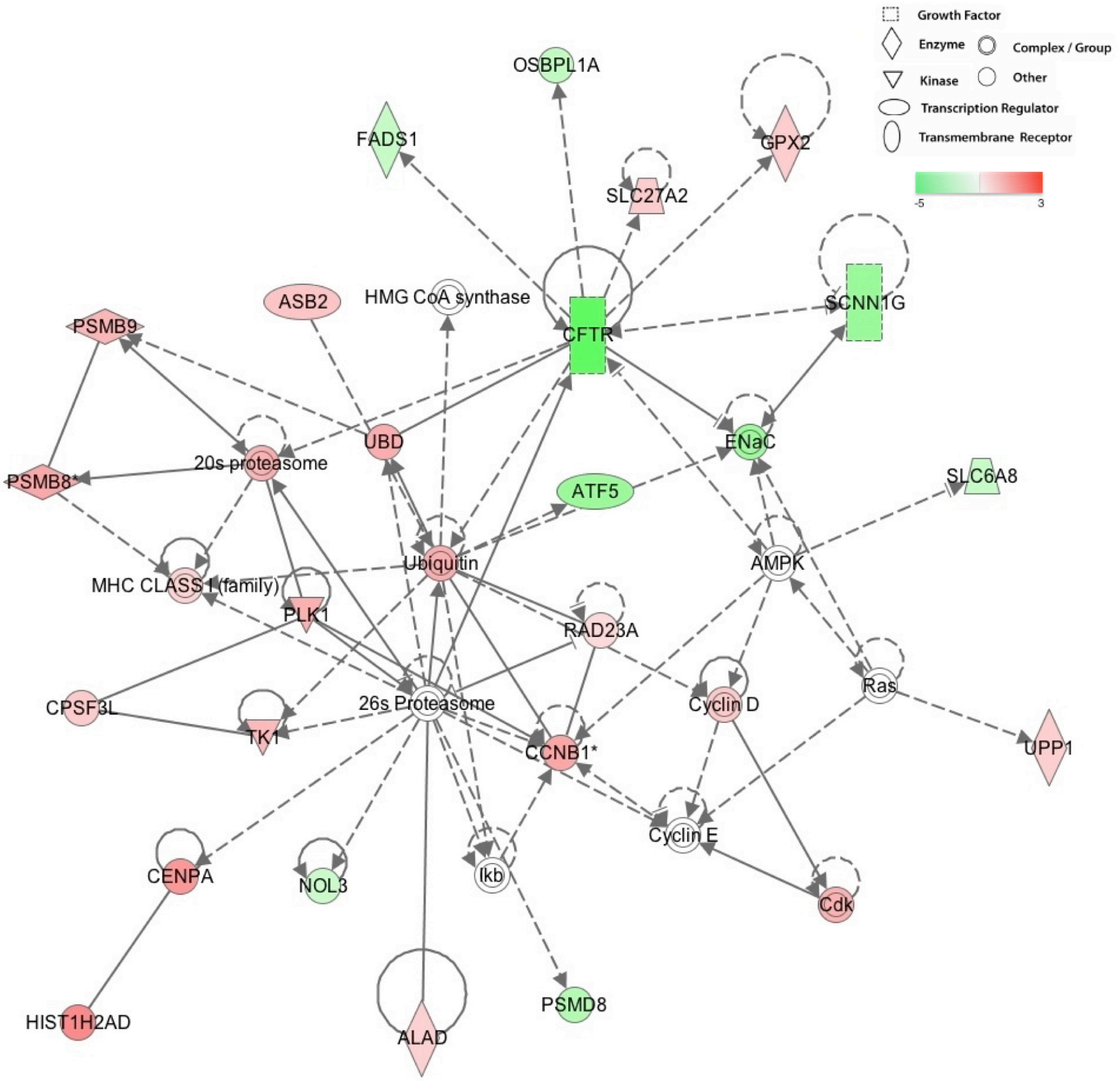

Fig. S3 Stress, transporters and DNA replication regulatory map. Generated by the Ingenuity pathway analysis of differentially expressed metabolic genes identified from the processed transcriptomic datasets between pBmpr1aKO and Control islets at 3 months.

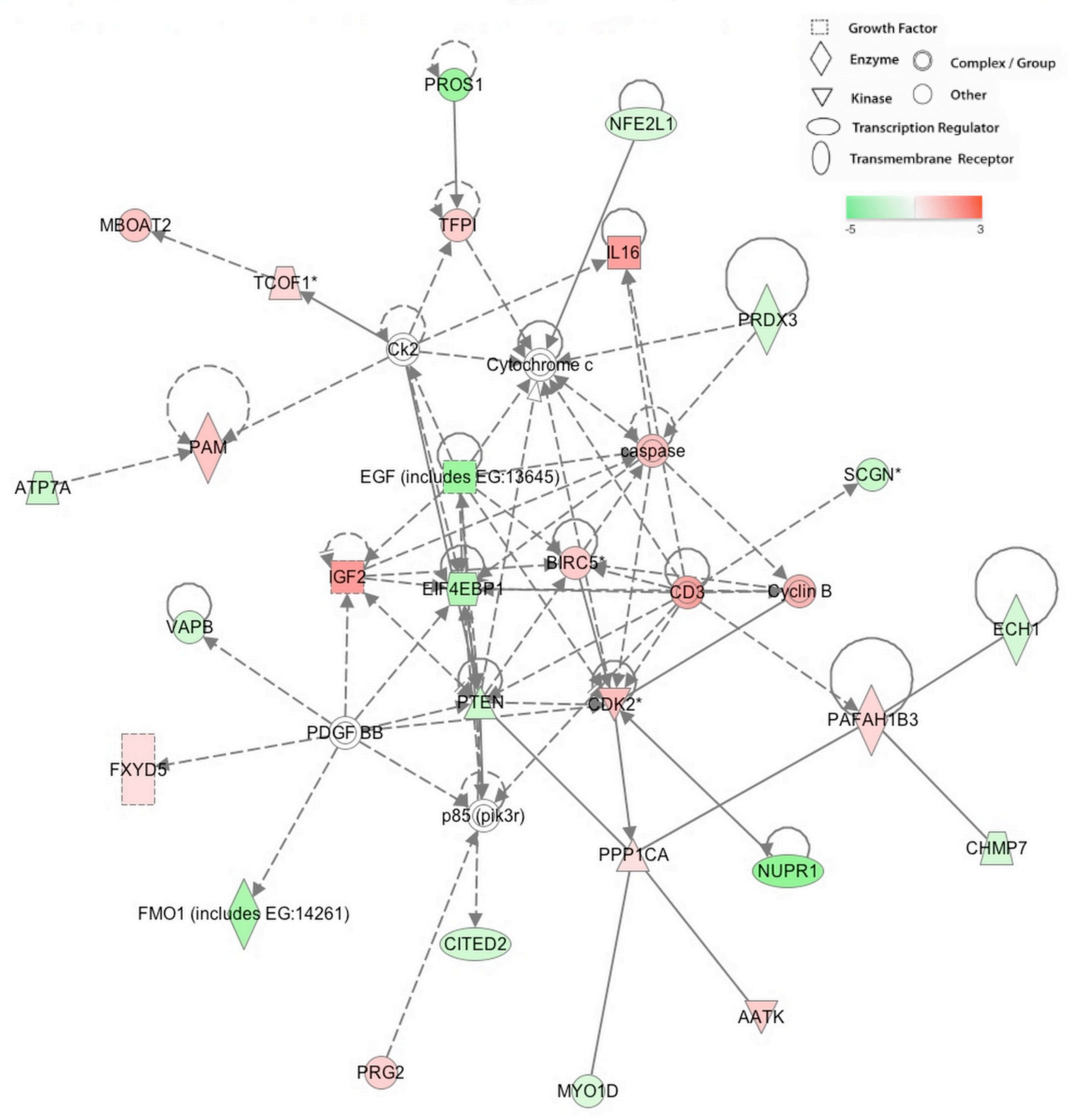

Fig. S4 Cell cycle and apoptosis regulatory map. Generated by the Ingenuity pathway analysis of differentially expressed metabolic genes identified from the processed genome-wide transcriptomic datasets between pBmpr1aKO and Control islets at 3 months.
